# Switchable Ultrathin Quarter-wave Plate in Terahertz Using Active Phase-change Metasurface

**DOI:** 10.1038/srep15020

**Published:** 2015-10-07

**Authors:** Dacheng Wang, Lingchao Zhang, Yinghong Gu, M. Q. Mehmood, Yandong Gong, Amar Srivastava, Linke Jian, T. Venkatesan, Cheng-Wei Qiu, Minghui Hong

**Affiliations:** 1 Department of Electrical and Computer Engineering, National University of Singapore, 4 Engineering Drive 3, 117576, Singapore; 2 Institute for Infocomm Research, 1 Fusionoplis Way, #21-01 Connexis, 138632, Singapore; 3 Department of Physics, National University of Singapore, 2 Science Drive 3, 117542, Singapore; 4 NUSNNI-NanoCore, National University of Singapore, 5A Engineering Drive 1, 117411, Singapore; 5 Materials Science and Engineering and Integrative Science and Engineering, National University of Singapore, 117456, Singapore

## Abstract

Metamaterials open up various exotic means to control electromagnetic waves and among them polarization manipulations with metamaterials have attracted intense attention. As of today, static responses of resonators in metamaterials lead to a narrow-band and single-function operation. Extension of the working frequency relies on multilayer metamaterials or different unit cells, which hinder the development of ultra-compact optical systems. In this work, we demonstrate a switchable ultrathin terahertz quarter-wave plate by hybridizing a phase change material, vanadium dioxide (VO_2_), with a metasurface. Before the phase transition, VO_2_ behaves as a semiconductor and the metasurface operates as a quarter-wave plate at 0.468 THz. After the transition to metal phase, the quarter-wave plate operates at 0.502 THz. At the corresponding operating frequencies, the metasurface converts a linearly polarized light into a circularly polarized light. This work reveals the feasibility to realize tunable/active and extremely low-profile polarization manipulation devices in the terahertz regime through the incorporation of such phase-change metasurfaces, enabling novel applications of ultrathin terahertz meta-devices.

Control of polarization, a fundamental physical property of electromagnetic (EM) waves, is manifested for a wide range of optical applications, using polarizers, half- and quarter-wave plates in different frequency regimes. Conventional methods for the polarization state control are based on birefringent materials, which support different phase delays along the two orthogonal axes. The main disadvantages of such methods include narrow-band of operating frequencies, bulky size and limited choice of materials. The situation is even worse in terahertz (THz) frequency regime (0.3 THz–3 THz), as THz wave interacts weakly with available materials in nature. For instance, the birefringence index Δn of quartz at 1 THz is around 0.048, which means that the thickness of quartz based THz quarter-wave plate needs to be at least a few millimeters^1^. Such a device is difficult to be integrated into ultra-compact THz optical systems. Expanding the working frequency of the birefringent materials based devices relies on multilayered designs, which further contributes to the device bulky^2^.

Metasurfaces, which are two-dimensional artificially engineered media, have enabled new approaches to manipulate the wavefront of EM waves. Composed of thin optical resonators of different materials and geometries, metasurfaces can control both the amplitude and phase of EM waves^3^. As demonstrated recently^4^, metasurfaces present unique properties, such as extremely short effective wavelength, abrupt phase change and strong chromatic dispersion, leading to many surprising phenomena. An intriguing property of metasurface is its ability to manipulate EM waves in a thin film of only nanoscale thickness, which promises potential for integration into ultra-compact optical systems. So far, researchers have demonstrated several extraordinary properties of metasurfaces, such as polarization conversion^5^,^6^,^7^,^8^,^9^,^10^, perfect absorption^11^,^12^, amplitude and phase modulation^13^,^14^,^15^,^16^,^17^,^18^,^19^,^20^,^21^,^22^,^23^,^24^,^25^. Particularly, flat lenses based on metasurfaces have become a milestone of the third-generation imaging devices^4^,^25^,^26^. Recently, metasurfaces-based birefringence has been demonstrated in THz range by using microscale resonators^27^,^28^. Due to the static response of the resonators, these metasurfaces work in a narrow frequency band. On the other hand, by stacking different layers of metasurfaces, the working frequency can be extended to a broadband^29^,^30^,^31^. However, this multilayer scheme needs an increased thickness and a more complicated fabrication process. To overcome this issue, Luo *et al.* established a theory for polarization controlling metasurface and presented the metasurface-assisted Fresnel’s equation^4^. Following this design principle, the chromatic dispersion of reflective metasurface was successfully utilized to achieve ultra-broadband polarization conversion^9^,^10^, demonstrating unprecedented performance compared with traditional devices. Luo has also unified the bandwidth-thickness limit^9^ of the perfect absorber and waveplate. It was shown that the thickness of broadband waveplates could be suppressed below the classic limit set by Max Planck almost a century ago^4^.

Metasurfaces integrated with active media enable the dynamic modulation of the optical properties without compromising the device thickness. Several active tuning media have been investigated, such as semiconductors^32^, phase change materials^33^,^34^,^35^ and MEMS^36^,^37^. In this work, we demonstrate a switchable ultrathin THz quarter-wave plate (QWP) by inserting vanadium dioxide (VO_2_) into metasurfaces. Through the phase transition, the conductivity of VO_2_ varies as much as almost four orders of magnitude, which makes VO_2_ a good platform for optical switching^38^. In our design, VO_2_ changes the effective length of resonators via the phase transition and the operating frequency of the QWP becomes switchable. At the working frequencies, the calculated ellipticities indicate a good polarization conversion. A Lorentz oscillator model is employed to analytically describe the performance of the THz QWP, which is in good agreement with the measurement and simulation. This switchable ultrathin THz QWP is applicable in compact tunable THz optical systems and this VO_2_ metasurface promises a new route for active micro/nano-photonic devices. Furthermore, this work could be extended to adaptive conformal polarization controlling metasurfaces, as inspired by the concept originally proposed in the work of Luo *et al.*^4^.

## Results and Discussion

**Figure 1:**
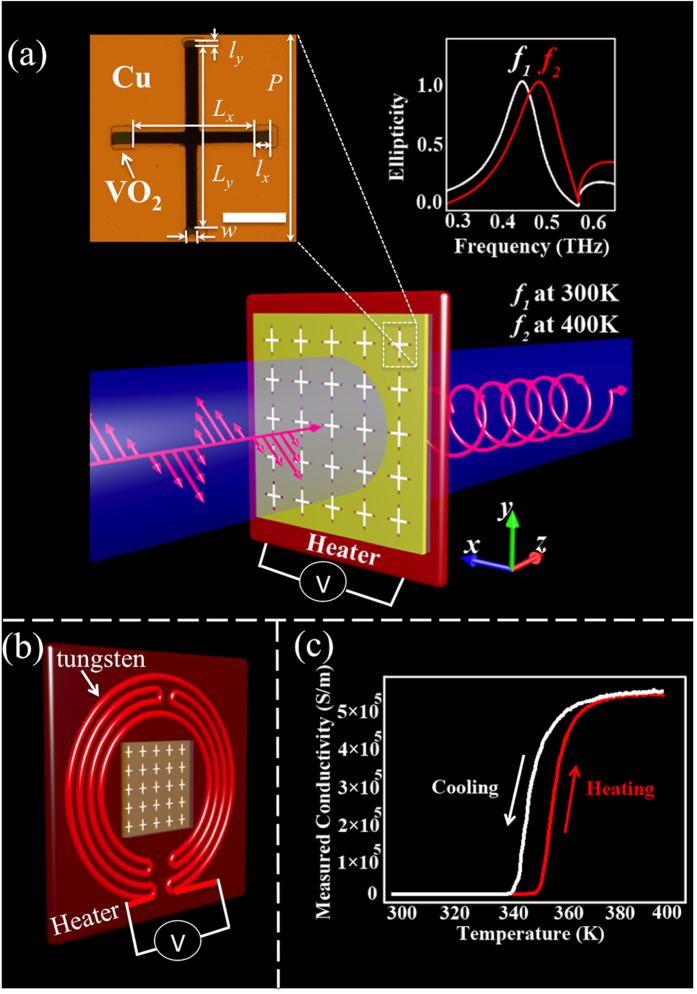
Switchable THz QWP design and fabrication results. (**a**) Experimental switching schematic of the THz QWP. A linear normal incident THz wave polarized at *θ* = 45° to the two slots is converted into a circularly polarized light. Through the VO_2_ phase transition controlled by a resistive heater, the operating frequency of the QWP can be switched between *f*_*1*_ = 0.468 THz and *f*_*2*_ = 0.502 THz. The top left inset is a microscope image of one unit cell in the fabricated samples. The scale bar is 50 μm. The following are the geometrical parameters: *P* = 150, *L*_*x*_ = 90, *L*_*y*_ = 124, *l*_*x*_ = 9, *l*_*y*_ = 5 and *w* = 9 μm, respectively. The top right inset is the simulated ellipticities of the output THz waves, indicating that at both *f*_*1*_ and *f*_*2*_ the output THz waves are circularly polarized. (**b**) Schematic backside view of the resistive heater with a square aperture (6 × 6 mm^2^) milled at the center to allow THz to pass through. (**c**) Measured electrical conductivity of fabricated VO_2_ films at different temperatures during the heating and the cooling cycles. The fabricated films exhibit stable electrical conductivity switching between 300 and 400 K during either the heating or the cooling cycles.

Figure 1a shows a schematic of the switchable QWP, which is composed of ultrathin asymmetric cross-shaped resonator arrays with VO_2_ pads inserted at the end of the cross-shaped resonators. The complementary metasurfaces present high transmission coefficients at the resonance frequencies due to the extraordinary optical transmission effect with specific phase delays^39^,^40^. It only allows the resonant EM wave to pass through, which eliminates the interference of the non-resonant EM wave. In our design, two slots in the QWP are perpendicular to each other with a slight difference in length. The fundamental resonance in each slot is able to present a maximum phase shift of 180° between the transmitted and the incident light. Therefore, birefringence can be introduced by controlling the length of the slots in the asymmetric cross-shaped resonators. The switching property of the QWP is controlled by a resistive heater as a proof of concept to manipulate the VO_2_ phase transition at different temperatures, which can also be realized by optical pumping^41^. When VO_2_ pads act as a semiconductor at 300 K, phase difference between two orthogonal slots can reach 90° at *f*_*1*_ = 0.468 THz, while the transmission coefficients in these two slots are the same. At this frequency, when the incident THz wave is polarized at *θ* = 45° to the two slots, the device operates as a QWP. Through the phase transition, free carriers in the VO_2_ pads increase, resulting in the rise of the electrical conductivity. The VO_2_ pads behave like a metal at 400 K. This leads to a shortened effective length of cross-shaped resonators and the QWP operates at *f*_*2*_ = 0.502 THz. Therefore, this THz QWP can switch its operating frequency between two states through the VO_2_ phase transition. The top right inset is the simulated ellipticities of the output THz wave at 300 and 400 K. It is observed that the ellipticites at *f*_*1*_ = 0.468 and *f*_*2*_ = 0.502 THz are close to 1, indicating a circular polarization of the output THz waves. The top left inset in Fig. 1a shows a microscope image of one unit cell in the fabricated metasurfaces.

The VO_2_ pads were fabricated by pulsed laser deposition (PLD) on a c-cut sapphire substrate using a metallic vanadium target in an oxidizing background^42^. The asymmetric cross-shaped resonators were patterned by photolithography and ion milling (see the methods). To ensure a full coverage between the metal film and VO_2_ pads, the dimensions of VO_2_ pads are slightly larger than the designed parameters. The total size of the fabricated sample is 1 × 1 cm^2^. Figure 1b shows a schematic backside view of the resistive heater with a square aperture (6 × 6 mm^2^) milled at the center. The curved wires are tungsten resistors used for heating, which are controlled by an external voltage source. The electrical conductivity of the deposited VO_2_ film was measured in van der Pauw geometry using Quantum design PPMS^42^. The VO_2_ film shows a sharp insulator-metal transition as shown in Fig. 1c. In the insulating state, the electrical conductivity reaches σ = 140 *S/m* at 300 K. In the high temperature metal phase, the electrical conductivity is relatively temperature-independent at σ = 5 × 10^5^ *S/m* at 400 K. The transition temperature during the heating cycle is about 353 K while it shifts to 342 K during cooling cycle with a hysteresis of 11 K.

A THz time domain spectroscope was used to perform the characterization (see the methods)^43^. The input and output polarizations of THz wave were controlled by two THz wire-grid polarizers. The QWP was measured with a normal incident THz wave polarized at *θ* = 45° to the two slots. The transmitted electric fields of THz wave were characterized along *x*- and *y*-axes, which were noted as *Ē*_*x*_ and *Ē*_*y*_. A sapphire substrate with transmitted electric fields *Ē*_*x*_*(ref)* and *Ē*_*y*_*(ref)* was tested as a reference. The transmission coefficients of the QWP were calculated as 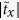 = *|Ē*_*x*_*/Ē*_*x*_*(ref)|* and 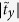  = *|Ē*_*y*_*/Ē*_*y*_*(ref)|*. The phase information of the THz wave along *x*- and *y*-axes were extracted by a fast Fourier transform, which were denoted as *φ*_*x*_ and *φ*_*y*_. The phase delay between *y*- and *x*-axes was *φ* = *φ*_*y*_ − *φ*_*x*_ = arg(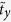) − arg(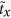). At temperatures above the VO_2_ phase transition (controlled by the heater), the transmission coefficients and phase delays were measured by the same approach described above.

**Figure 2:**
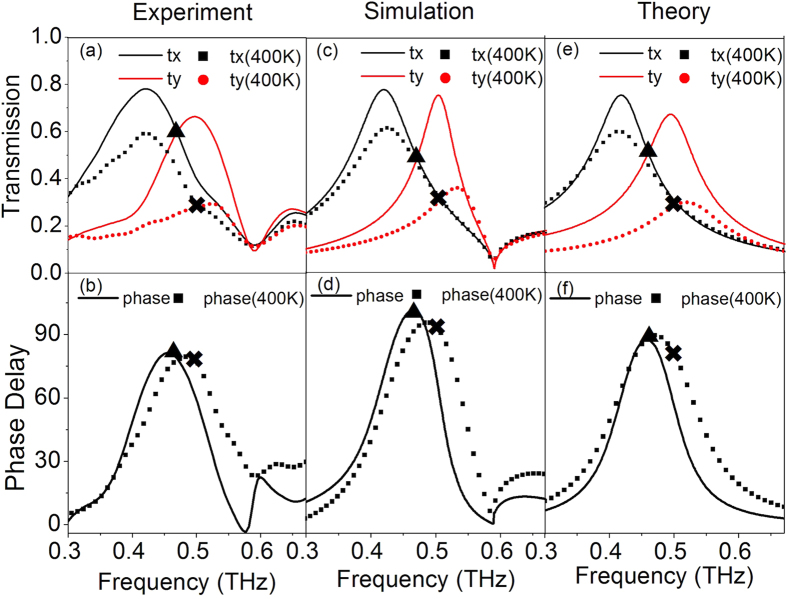
Performance of the switchable THz QWP. (**a**) Measured transmission spectra along two slots at 300 K (solid line) and 400 K (dot line). (**b**) Measured phase difference between y- and x-axes at 300 K (solid line) and 400 K (dot line). The inserted triangle indicates at 300 K, the transmission coefficients along two axes are the same, while their phase difference is close to 90°. The marked cross presents similar results at 400 K. (**c**) Numerically simulated transmission spectra with the corresponding phase delay (**d**) based on different measured VO_2_ electrical conductivities. (**e**) Analytical fitted transmission spectra and (**f**) the corresponding phase delays.

Figure 2a,b show the measured transmission coefficients and phase delays of THz QWP before and after the VO_2_ phase transition. At 300 K, the QWP presents a transmission coefficient of 0.59 at 0.468 THz and a phase delay of 80° between *y*- and *x*-axes. This means the incident linearly polarized THz wave is converted into a circularly polarized wave. When the QWP is heated to 400 K, the VO_2_ phase transition changes the performance of the QWP. As it can be seen, a transmission coefficient of 0.28 at 0.502 THz and a phase delay of 75° between *y*- and *x*-axes are obtained. Therefore, the THz QWP is able to switch its operating frequency with a switching range of 34 GHz. The numerical simulation and analytical model fitting results are shown in Fig. 2c–f, which will be discussed in the simulation and analytical modeling sections. The experimental phase delays at both 300 and 400 K are smaller than 90°, indicating that the output THz wave is not a perfect circularly polarized light. This is due to the size fluctuation in the fabrication process and the damping effect in the fabricated samples. To experimentally compensate the phase difference and obtain a perfect circularly polarized output THz wave, the optimized phase delay in the simulation should be larger than 90°.

In order to further investigate the polarization state of output THz wave and its relation to the VO_2_ phase transition, the transmission coefficients and phase delays of the THz QWP at different temperatures were measured by the THz-TDS system and the Stokes parameters were calculated using the following equations^6^,^29^:


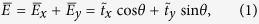



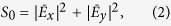



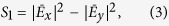



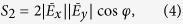



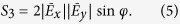


**Figure 3:**
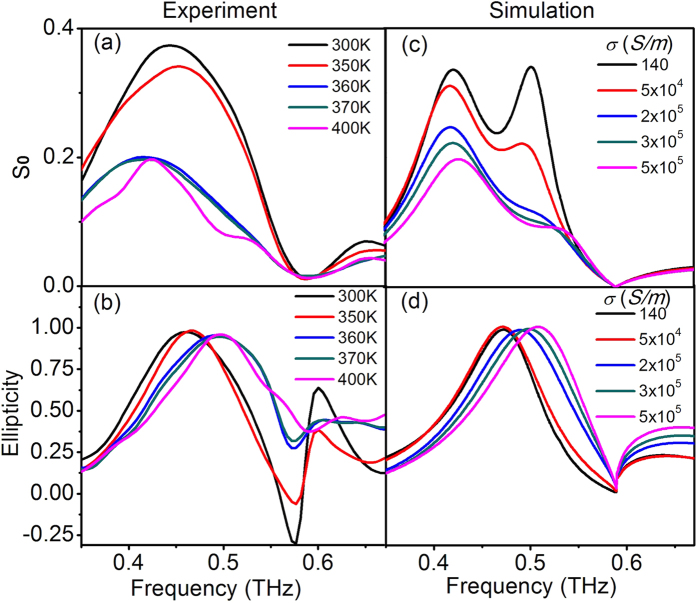
Performance of the THz QWP at different temperatures. (**a**) Calculated Stokes parameter *S*_*0*_ with respect to different temperatures based on THz-TDS measured results, indicating that the output power decreases when the temperature increases. (**b**) Measured ellipticities of the output THz wave at different temperatures, indicating the operation frequencies switching of the output circularly polarized THz wave. (**c**) Numerically simulated Stokes parameter *S*_*0*_ with respect to different conductivities of VO_2_ and (**d**) the corresponding ellipticities of the output THz wave.

Figure 3a shows the measured *S*_*0*_ parameters at different temperatures, which indicate the power of the output THz wave. It is observed that when the temperature increases from 300 to 400 K, the output power decreases. This is attributed to loss in the VO_2_ pads. At 300 K, the VO_2_ pads behave as a semiconductor and the corresponding loss is low. When the temperature increases, free carries in the VO_2_ pads increase, leading to a high damping loss and small output power. From Fig. 2a and 3a, we can observe asymmetric transmitted peak amplitudes of 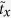 and 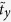. When the conductivity of VO_2_ increases, the resonance frequency of 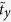 shifts to a higher frequency, which is closer to the wood’s anomaly at 0.59 THz. The peak amplitude of 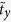 becomes smaller, which is shown in Fig. 2a. Another contribution to this asymmetric peak transmission is the different sizes of VO_2_ pads. As shown in Fig. 1a, the size of VO_2_ pads along the *x*-axis is much larger than that along *y*-axis. This leads to different VO_2_ damping losses for 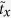 and 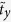. The polarization state of the output THz wave can be described by ellipticity, which is defined as χ = *S*_*3*_/*S*_*0*_. When χ equals to either 1 or −1, the output THz wave is circularly polarized. Figure 3b shows the ellipticity of the output THz wave at different temperatures. At 300 K, the ellipticity of the output THz wave is around 0.98 at 0.468 THz. At 400 K, the ellipticity is around 0.97 at 0.502 THz. Between 300 and 400 K, the ellipticity is close to 1 with the operating frequency switching from 0.468 to 0.502 THz. This indicates that the output THz wave is circularly polarized at different temperatures. The numerical simulation results are shown in Fig. 3c,d, which will be discussed in the following section.

To confirm the performance of our designed THz QWP, numerical simulation was carried out by using commercial software CST Microwave Studio. In the simulation, a frequency-domain solver with a unit cell boundary condition was used to calculate the transmission coefficients and phase delays. The sapphire substrate was modeled as a lossless dielectric with a dielectric constant of *ε*_sub_ = 11.5 and the copper film was simulated as a lossy metal with a conductivity of σ = 5.8 × 10^7^ *S/m*. A variable conductivity of VO_2_ was assumed to simulate the phase transition effect in the VO_2_ pads. The optical constant of VO_2_ in THz range was described by Drude Model as follows^44^,^45^:


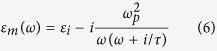



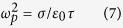


where *ε*_*m*_ was the dielectric function, *ε*_*i*_ was the dielectric constant as 9, *ω*_*p*_ was the plasma frequency*, σ* was the measured dc conductivity *and τ* was the relaxation time as 2.27 f*s.* The simulated transmission spectra and phase delays are shown in Fig. 2c,d. When the conductivity of VO_2_ is at σ = 140 S/m at 300 K, the QWP presents a transmission coefficient of 0.49 at 0.471 THz with a phase difference of 100° between y- and x-axes. When the conductivity of VO_2_ is at σ = 5 × 10^5^ S/m at 400 K, the transmission coefficient is 0.30 at 0.508 THz and the phase difference is 88°. This means that the output THz waves at both 300 and 400 K are circularly polarized. At 0.59 THz, the wood’s anomaly in the simulation occur, which can be calculated as 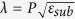, where *P* is the periodicity of the resonators and *ε*_sub_ is the dielectric constant of sapphire substrate^46^. The performance of the QWP at different temperatures is simulated, which is shown in Fig. 3c and d. When the conductivity of VO_2_ increases from 140 to 5 × 10^5^ *S/m*, the output power *S*_*0*_ decreases and the ellipticity is around 1 with a shift of the operating frequency. This is consistent with our THz-TDS measured results. The slight difference between the experimental and simulation results may be due to inadvertent dimensional variation during the fabrication. Experimentally, the resonance peaks are broader than those in the simulation. Another reason for the broadening of the resonance peaks might be the temperature gradient on the sample surface during heating in the THz-TDS system. This confirms that the experimental phase delay is smaller than the simulated results, which can be further optimized to achieve a perfect linear-to-circular polarization conversion.

To further elucidate the physics behind the designed QWP, a Lorentz oscillator model was introduced to analyze the performance of the THz QWP and the phase transition effect in the VO_2_ pads, following the dispersion model for anisotropic metasurface given by Luo *et al.*^9^. The incident electromagnetic wave can excite resonance modes inside the slots in the QWP, which is similar to rod antennas based on Babinet’s principle^39^,^40^,^47^. When the incident THz wave is polarized along *x*-axis, the corresponding transmitted electric field can be depicted as:


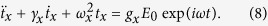


Similarly, for *y*-axis polarized incident THz wave, the transmitted electric field is modeled as:


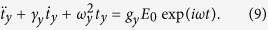


*t*_*x*_*, t*_*y*_*, γ*_*x*_and *γ*_*y*_ are transmitted electric fields and damping rates of two orthogonal resonance modes in the cross-shaped resonators. ω_*x*_ and ω_*y*_ are the corresponding resonance frequencies of two modes. *g*_*x*_ and *g*_*y*_ are geometric factors in the cross-shaped resonators. *E*_*0*_exp*(iωt)* is the electric field of the incident THz wave. By solving Equations (8) and (9), the analytical solutions for transmission coefficients *t*_*x*_ and *t*_*y*_with the corresponding phase distributions *φ*_*x*_ and *φ*_*y*_ can be obtained.

**Figure 4:**
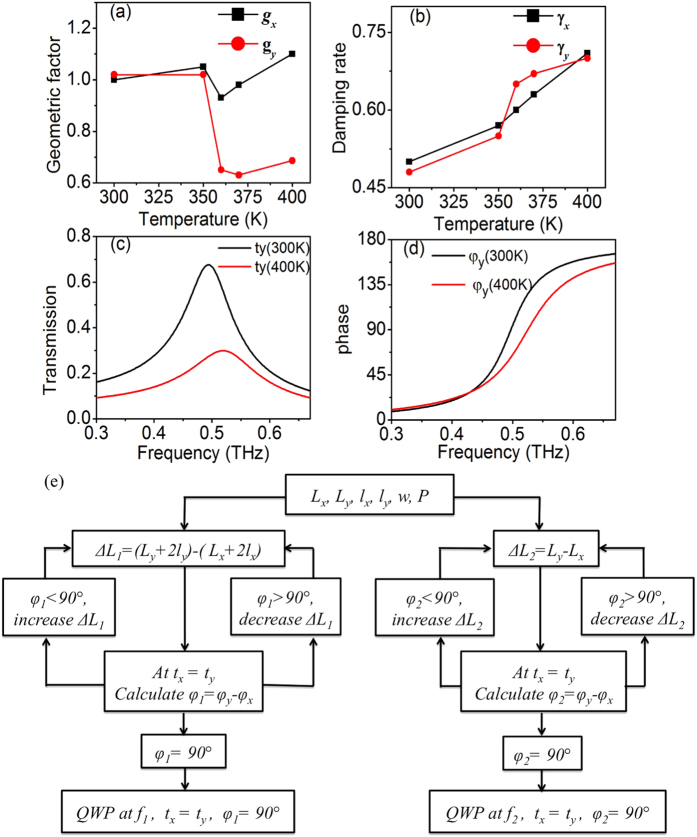
Temperature dependent behaviors of the fitting parameters and the flowchart for QWP design. (**a**) Analytical fitted geometric factors and (**b**) damping rates at different temperatures. (**c**) Fitted transmission spectra along y-axis at 300 and 400 K. (**d**) Fitted phase distributions along y-axis at 300 and 400 K. (**e**) A QWP design flowchart to optimize the parameters of the phase-change metasurfaces.

With a proper parameter fitting based on the experimental results, the transmission spectra and phase delays of the THz QWP are plotted in Fig. 2e,f, which is in good agreement with the measurement and simulation. The fitted geometric factor *g* and damping rate *γ* at different temperatures are plotted in Fig. 4a,b. It is observed that when the VO_2_ pads go through the phase transition, the geometric factors decrease dramatically, indicating a weak coupling between the incident THz wave and the VO_2_ metasurfaces. When the conductivity of VO_2_ further increases to its maximum point, the geometric factor slightly increases. The difference of *g*_*x*_ and *g*_*y*_ indicates different losses for 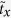 and 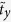, which are consistent with the measured and simulated results. The damping rates plotted in Fig. 4b show that both *γ*_*x*_and *γ*_*y*_ increase with temperature. To illustrate the correlation of the fitting parameters and the performance of QWP, the fitted transmission coefficients and phase distributions at 300 and 400 K along *y*-axis are plotted in Fig. 4c,d. It is observed that the decrease of geometric factors correlate with the decrease of transmission coefficients. The increase of damping rates leads to a large resonance bandwidth as the spectral phase dispersion tends to be flat.

Based on these observations, a flowchart for optimization of parameters in such VO_2_ metasurface-based QWP is presented in Fig. 4e. For simplicity, the periodicity *P* and the width *w* remain unchanged and we focus on optimizing the length of copper resonators and VO_2_ pads. For the 300 K case, we define the length difference between *y-* and *x-*axes as Δ*L*_*1*_ = (*L*_*y*_ + 2*l*_*y*_) − (*L*_*x*_ + 2*l*_*x*_). With any initial parameter of *L*_*x*_, *L*_*y*_, *l*_*x*_ and *l*_*y*_, we can calculate the transmission spectra and phase delays. If at *t*_*x*_ = *t*_*y*_, the phase difference *φ*_*1*_ = *φ*_*y*_ − *φ*_*x*_ equals to 90°, the operating frequency of the QWP at *f*_*1*_ is obtained. Otherwise, Δ*L*_*1*_ needs to be increased or decreased according to the flowchart. Similarly, for the case at 400 K, we define the length difference between *y-* and *x*-axes as Δ*L*_*2*_ = *L*_*y*_ − *L*_*x*_ and optimize the parameters to obtain the operating frequency of *f*_*2*_. Based on the variation trend of the geometric factors, the transmission coefficients of QWP at *f*_*2*_ are smaller than those at *f*_*1*_. Due to the increase of damping rates, the resonances tend to be broad and Δ*L*_*2*_should be larger than Δ*L*_*1*_ to maintain a phase delay of 90°. Therefore, the flowchart in Fig. 4e presents the optimization process for the VO_2_ metasurface-based QWP. A similar approach can be applied to other phase-change metasurfaces for both amplitude and phase manipulation.

## Conclusion

In summary, by hybridizing metasurfaces with VO_2_, we have experimentally demonstrated an ultrathin switchable THz QWP with a switching range of 34 GHz. The inserted VO_2_ is able to change the effective length of the metal resonators in the metasurface through the phase transition. At 300 K, VO_2_ behaves like a semiconductor and the THz QWP operates at 0.468 THz. While at 400 K, VO_2_ acts as a metal and the operating frequency of the QWP is switched to 0.502 THz. The Stokes parameters of the output THz wave calculated at different temperatures indicate that the output wave is circularly polarized. The damping loss in VO_2_ and the wood’s anomaly lead to the decrease of the transmission coefficients at a high temperature. The simulation and analytical fitted results are in good agreement with the measured results. The fitted geometric factors and damping rates analytically illustrate the correlation of VO_2_ phase transition and the performance of the THz QWP. This switchable phase-change metasurface promises a new route for active THz polarization manipulation devices and can be applied to other ultrathin tunable meta-devices.

## Methods

### Fabrication

The THz QWP was fabricated on a 1 × 1 cm^2^ c-cut sapphire single crystal substrate. A commercial vanadium single crystal (100) oriented metal target with 5N purity (from Goodfellow) was used for the VO_2_ thin film growth. A 248 nm KrF excimer laser (pulse duration 20 ns) at a rate of 5 Hz and a laser fluence of 2 J/cm^2^ was used at the target. The deposition conditions were optimized (substrate temperature of 500 °C at a background oxygen pressure of 1 mTorr) to get a sharp metal-insulator transition. After 40,000 pulses deposition, the oxygen pressure was immediately increased to 5 mTorr and the VO_2_ film was annealed under this pressure for one hour at 500 °C. Then the sample was cooled down at a rate of 10 °C/min to room temperature. A step surface profiler was used to measure the thickness of the VO_2_ film, which was around 200 nm. The designed VO_2_ pads were patterned by photolithography. The VO_2_ area without protection from photoresist was etched away by ion milling. After a second photolithography, a 10 nm thick chromium film was coated on the sapphire substrate as an adhesion layer and a 200 nm thick copper film was deposited on the samples by a thermal evaporator (Edwards Auto 306), followed by a lift-off process to obtain the designed patterns.

### Characterization

The performance of the QWP was tested in a THz time domain spectroscope. In this system, a femtosecond laser (10 fs, center wavelength 800 nm, repetition rate 80 MHz) was used to pump low-temperature-grown GaAs-based photoconductive antennas in the THz emitter and detector for THz wave generation and detection. Two wire-grid THz polarizers mounted on the rotation stages were positioned in front of the THz emitter and detector to control the polarization of the input and output THz waves. The system was sealed in a box with pure N_2_ gas to minimize the THz wave loss. A resistive heater with a square aperture (6 × 6 mm^2^) milled at the center was used to heat the samples and control the insulator-metal phase transition temperature of VO_2_. The temperature of the samples was monitored in real time by an infrared camera (FLIR Systems i60) to monitor the VO_2_ phase transition.

## Additional Information

**How to cite this article**: Wang, D. *et al.* Switchable Ultrathin Quarter-wave Plate in Terahertz Using Active Phase-change Metasurface. *Sci. Rep.* **5**, 15020; doi: 10.1038/srep15020 (2015).

## References

[b1] Masson J. B. & Gallot G. Terahertz achromatic quarter-wave plate. Opt. Lett. 31, 265–267 (2006). 10.1364/ol.31.000265 16441051

[b2] Chen Z. C., Gong Y. D., Dong H., Notake T. & Minamide H. Terahertz achromatic quarter wave plate: Design, fabrication, and characterization. Opt. Commun. 311, 1–5 (2014).

[b3] Kildishev A. V., Boltasseva A. & Shalaev V. M. Planar Photonics with Metasurfaces. Science 339, 1232009 (2013). 23493714 10.1126/science.1232009

[b4] Luo X. Principles of electromagnetic waves in metasurfaces. Sci. China: Phys., Mech. Astron. 58, 09421 (2015).

[b5] Cong L. Q. *et al.* A perfect metamaterial polarization rotator. Appl. Phys. Lett. 103, 171107 (2013).

[b6] Wang D. C., Gu Y. H., Gong Y. D., Qiu C. W. & Hong M. H. An ultrathin terahertz quarter-wave plate using planar babinet-inverted metasurface. Opt. Express 23, 11114–11122 (2015). 25969207 10.1364/OE.23.011114

[b7] Hao J. M. *et al.* Optical metamaterial for polarization control. Phys. Rev. A 80, 023807 (2009).

[b8] Ma X. L. *et al.* Dual-band asymmetry chiral metamaterial based on planar spiral structure. Appl. Phys. Lett. 101, 161901 (2012).

[b9] Pu M. B. *et al.* Anisotropic meta-mirror for achromatic electromagnetic polarization manipulation. Appl. Phys. Lett. 102, 131906 (2013).

[b10] Guo Y. H. *et al.* Dispersion management of anisotropic metamirror for super-octave bandwidth polarization conversion. Sci. Rep. 5, 8434 (2015). 25678280 10.1038/srep08434 PMC4326699

[b11] Shen X. P. & Cui T. J. Photoexcited broadband redshift switch and strength modulation of terahertz metamaterial absorber. J. Opt. 14, 114012 (2012).

[b12] Pu M. B. *et al.* Ultrathin broadband nearly perfect absorber with symmetrical coherent illumination. Opt. Express 20, 2246–2254 (2012). 22330464 10.1364/OE.20.002246

[b13] Lin D. M., Fan P. Y., Hasman E. & Brongersma M. L. Dielectric gradient metasurface optical elements. Science 345, 298–302 (2014). 25035488 10.1126/science.1253213

[b14] Wang D. C., Qiu C. W. & Hong M. H. Coupling effect of spiral-shaped terahertz metamaterials for tunable electromagnetic response. Appl. Phys. A 115, 25–29 (2014).

[b15] Wang D. C., Huang Q., Qiu C. W. & Hong M. H. Selective excitation of resonances in gammadion metamaterials for terahertz wave manipulation. Sci. China: Phys., Mech. Astron. 58, 084201 (2015).

[b16] Pu M. B. *et al.* Spatially and spectrally engineered spin-orbit interaction for achromatic virtual shaping. Sci. Rep. 5, 9822 (2015). 25959663 10.1038/srep09822 PMC4426594

[b17] Liu L. X. *et al.* Broadband metasurfaces with simultaneous control of phase and amplitude. Adv. Mater. 26, 5031–5036 (2014). 24863731 10.1002/adma.201401484

[b18] Yang Y. M., Kravchenko I. I., Briggs D. P. & Valentine J. All-dielectric metasurface analogue of electromagnetically induced transparency. Nat. Commun. 5, 5753 (2014). 25511508 10.1038/ncomms6753

[b19] Gu J. Q. *et al.* Active control of electromagnetically induced transparency analogue in terahertz metamaterials. Nat. Commun. 3, 1151 (2012). 23093188 10.1038/ncomms2153

[b20] Estakhri N. M. & Alu A. Ultra-thin unidirectional carpet cloak and wavefront reconstruction with graded metasurfaces. IEEE Antenn. Wirel. Pr. Lett. 13, 1775–1778 (2014).

[b21] Huang L. L. *et al.* Three-dimensional optical holography using a plasmonic metasurface. Nat. Commun. 4, 2808 (2013).

[b22] Liang G. *et al.* Squeezing Bulk Plasmon Polaritons through Hyperbolic Metamaterials for Large Area Deep Subwavelength Interference Lithography. Adv. Opt. Mater. 10.1002/adom.201400596 (2015).

[b23] Gao P. *et al.* Enhancing aspect profile of half-pitch 32 nm and 22 nm lithography with plasmonic cavity lens. Appl. Phys. Lett. 106, 093110 (2015).

[b24] Yu N. F. *et al.* Light propagation with phase discontinuities: Generalized laws of reflection and refraction. Science 334, 333–337 (2011). 21885733 10.1126/science.1210713

[b25] Pu M. B. *et al.* Broadband anomalous reflection based on gradient low-Q meta-surface. AIP Adv. 3, 052136 (2013).

[b26] Zhang X. Q. *et al.* Broadband terahertz wave deflection based on C-shape complex metamaterials with phase discontinuities. Adv. Mater. 25, 4567–4572 (2013). 23787976 10.1002/adma.201204850

[b27] Wiesauer K. & Jordens C. Recent Advances in Birefringence Studies at THz Frequencies. J. Infrared Millim. Te. 34, 663–681 (2013).

[b28] Strikwerda A. C. *et al.* Comparison of birefringent electric split-ring resonator and meanderline structures as quarter-wave plates at terahertz frequencies. Opt. Express 17, 136–149 (2009). 19129881 10.1364/oe.17.000136

[b29] Cong L. Q. *et al.* Highly flexible broadband terahertz metamaterial quarter-wave plate. Laser Photonics Rev. 8, 626–632 (2014).

[b30] Grady N. K. *et al.* Terahertz Metamaterials for Linear Polarization Conversion and Anomalous Refraction. Science 340, 1304–1307 (2013). 23686344 10.1126/science.1235399

[b31] Fan R. H. *et al.* Freely tunable broadband polarization rotator for terahertz waves. Adv. Mater. 27, 1201–1206 (2015). 25545177 10.1002/adma.201404981

[b32] Zhang S. *et al.* Photoinduced handedness switching in terahertz chiral metamolecules. Nat. Commun. 3, 942–948 (2012). 22781755 10.1038/ncomms1908

[b33] Chen H. T. *et al.* Active terahertz metamaterial devices. Nature 444, 597–600 (2006). 17136089 10.1038/nature05343

[b34] Ma X. L. *et al.* An Active Metamaterial for Polarization Manipulating. Adv. Opt. Mater. 2, 945–949 (2014).

[b35] Chen Y. G. *et al.* Engineering the Phase Front of Light with Phase-Change Material Based Planar lenses. Sci. Rep. 5, 8660 (2015). 25726864 10.1038/srep08660 PMC4345347

[b36] Zhu W. M. *et al.* A MEMS tunable metamaterial filter. Proc. IEEE Micr. Elect. **Jan 24–28**, 196–199 (2010).

[b37] Ma F. S., Lin Y. S., Zhang X. H. & Lee C. Tunable multiband terahertz metamaterials using a reconfigurable electric split-ring resonator array. Light Sci. Appl. 3, e171 (2014).

[b38] Zhu Y. H., Zhao Y., Holtz M., Fan Z. Y. & Bernussi A. A. Effect of substrate orientation on terahertz optical transmission through VO2 thin films and application to functional antireflection coatings. J. Opt. Soc. Am. B 29, 2373–2378 (2012).

[b39] Falcone F. *et al.* Babinet principle applied to the design of metasurfaces and metamaterials. Phys. Rev. Lett. 93, 197401 (2004). 15600876 10.1103/PhysRevLett.93.197401

[b40] Zalkovskij M. *et al.* Optically active Babinet planar metamaterial film for terahertz polarization manipulation. Laser Photonics Rev. 7, 810–817 (2013).

[b41] Cavalleri A. *et al.* Femtosecond structural dynamics in VO2 during an ultrafast solid-solid phase transition. Phys. Rev. Lett. 87, 237401 (2001). 11736474 10.1103/PhysRevLett.87.237401

[b42] Srivastava A. *et al.* Coherently coupled ZnO and VO2 interface studied by photoluminescence and electrical transport across a phase transition. Appl. Phys. Lett. 100, 241907 (2012).

[b43] Dong H., Gong Y. D., Paulose V. & Hong M. H. Polarization state and Mueller matrix measurements in terahertz-time domain spectroscopy. Opt. Commun. 282, 3671–3675 (2009).

[b44] Jepsen P. U. *et al.* Metal-insulator phase transition in a VO2 thin film observed with terahertz spectroscopy. Phys. Rev. B 74, 1–9 (2006).

[b45] Mandal P., Speck A., Ko C. & Ramanathan S. Terahertz spectroscopy studies on epitaxial vanadium dioxide thin films across the metal-insulator transition. Opt. Lett. 36, 1927–1929 (2011). 21593938 10.1364/OL.36.001927

[b46] Kravets V. G., Schedin F. & Grigorenko A. N. Extremely narrow plasmon resonances based on diffraction coupling of localized plasmons in arrays of metallic nanoparticles. Phys. Rev. Lett. 101, 087403 (2008). 18764660 10.1103/PhysRevLett.101.087403

[b47] Choe J. H., Kang J. H., Kim D. S. & Park Q. H. Slot antenna as a bound charge oscillator. Opt. Express 20, 6521–6526 (2012). 22418535 10.1364/OE.20.006521

